# Research Progress on Methods for Improving the Stability of Non-Destructive Testing of Agricultural Product Quality

**DOI:** 10.3390/foods13233917

**Published:** 2024-12-04

**Authors:** Sai Xu, Hanting Wang, Xin Liang, Huazhong Lu

**Affiliations:** 1 Institute of Facility Agriculture, Guangdong Academy of Agricultural Sciences, Guangzhou 510640, China; liangxin@gdaas.cn; 2 School of Life Sciences, South China Normal University, Guangzhou 510631, China; hantingw316@outlook.com; 3 Guangdong Academy of Agricultural Sciences, Guangzhou 510640, China

**Keywords:** non-destructive testing, agriculture product, system stability, algorithm improvement, hardware system improvement

## Abstract

Non-destructive testing (NDT) technology is pivotal in the quality assessment of agricultural products. In contrast to traditional manual testing, which is fraught with subjectivity, inefficiency, and the potential for sample damage, NDT technology has gained widespread application due to its advantages of objectivity, speed, and accuracy, and it has injected significant momentum into the intelligent development of the food industry and agriculture. Over the years, technological advancements have led to the development of NDT systems predicated on machine vision, spectral analysis, and bionic sensors. However, during practical application, these systems can be compromised by external environmental factors, the test samples themselves, or by the degradation and noise interference inherent in the testing equipment, leading to instability in the detection process. This instability severely impacts the accuracy and efficiency of the testing. Consequently, refining the detection methods and enhancing system stability have emerged as key focal points for research endeavors. This manuscript presents an overview of various prevalent non-destructive testing methodologies, summarizes how sample properties, external environments, and instrumentation factors affect the stability of testing in practical applications, organizes and analyzes solutions to enhance the stability of non-destructive testing of agricultural product quality based on current research, and offers recommendations for future investigations into the non-destructive testing technology of agricultural products.

## 1. Introduction

In contemporary agricultural production, the expeditious and objective assessment of agricultural product quality constitutes an essential procedure, which plays a crucial role in maintaining the high standards of marketed agricultural products [1,2]. Non-destructive testing (NDT) technology has risen as an efficacious solution and is extensively applied in the qualitative evaluation of agricultural commodities, encompassing fruits and vegetables [3,4,5], meat [6,7,8], and beverages such as tea and wine [9,10], due to its non-invasive nature, high efficiency, and the precision of its outcomes. Over recent years, with the evolution of computer science, optoelectronics, sensor technology, and other technical disciplines, NDT has transitioned from a singular, target-specific methodology to a more holistic model that integrates diverse modalities and technical approaches. Online NDT systems have reached a level of maturity over time. The stability of detection is key to ensuring the reliability and consistency of results, and it is an important guarantee for achieving quality control and enhancement of agricultural products. It necessitates that the detection system sustains its robustness and repeatability throughout its application lifespan, which is particularly evident in the instruments’ resilience to external perturbations, the algorithms’ resistance to anomalous data, and the precision of the detection results. Nonetheless, the detection system may encounter various sources of error throughout its operational process, which can be categorized into the influence of the sample itself, environmental factors, and the impact of instrumentation, among others. Linear drift in spectroscopic and electronic nose systems due to temperature and humidity fluctuations, external noise interfering with the acquisition of acoustic vibration signals, and the randomness of sample placement affecting the capture of sample characteristics are examples of interference factors that can severely compromise detection performance. Consequently, it is imperative to implement strategies to mitigate these interferences.

In the quest to enhance the stability of non-destructive testing for assessing the quality of agricultural products, researchers have focused on optimizing various components of the detection system, including sample conditions, instrumental hardware, environmental factors, and algorithmic proficiency, with the aim of reducing errors across the entire analytical process. They have developed correction and generalization algorithms for the models, enhanced the structure and performance of hardware, and integrated rapidly evolving deep learning and multi-source data fusion technologies into the algorithmic models, resulting in notable performance enhancements. These studies have not only bolstered the adaptability and robustness of the employed NDT methods under diverse environmental conditions, but also introduced novel solutions for the precise evaluation of agricultural product quality, thereby enhancing detection efficiency and precision. While the existing research has significantly improved stability under specific conditions, several challenges persist. These include the effective enhancement of instrumental hardware and the challenge of transitioning models from controlled laboratory settings to real-world production environments for online detection while maintaining stable performance. These issues remain pressing and require resolution.

This manuscript provides a comprehensive review of the prevalent non-destructive testing (NDT) technologies utilized in the context of agricultural product quality. It explores the underlying causes of instability within NDT systems and deliberates on strategies designed to enhance stability, tailored to diverse technological emphases. Additionally, this article scrutinizes potential research directions, emerging trends, and forthcoming challenges in the evolution of NDT model performance, leveraging current technological advancements. The objective is to furnish valuable references and insights for researchers and practitioners in the field of agricultural product non-destructive testing.

## 2. Non-Destructive Testing Technology of Agricultural Products

### 2.1. Machine Vision

Machine vision, synonymous with computer vision, possesses the capability to extract meaningful information and features from visual signals such as images or videos for analysis and understanding, similar to the human visual system. A typical machine vision system encompasses two primary modules: image information acquisition and image information processing. This system can be further delineated into low-level processing (encompassing image capture and preprocessing), mid-level processing (featuring feature extraction and segmentation), and high-level processing (involving pattern recognition and decision-making) [11]. In the realm of image information acquisition, the image sensor is paramount, predominantly employing cameras and other hardware to digitize the necessary images. Beyond this, computerized tomography (CT), magnetic resonance (MR), and remote sensing are also prevalent techniques for acquiring images [12]. Image information processing is tasked with enhancing image quality by eliminating noise, rectifying distortions, and transforming visual data into discrete features through sophisticated feature extraction techniques [13]. Pattern recognition, conversely, is concerned with the qualitative analysis for classification and identification. The field has witnessed a steady progression and refinement from traditional machine learning algorithms, such as clustering [14], support vector machines [15], and decision trees [16], to the current state-of-the-art deep learning algorithms that leverage neural networks. Machine vision technology has become relatively mature in aspects of appearance inspection such as shape classification, defect detection [3], and quality assessment [17], and it can also be combined with various other methods to evaluate the internal quality of agricultural products. Furthermore, Sun et al. [18] and V. Narendra et al. [19] have provided detailed reviews on computer vision and pattern recognition methods for agricultural product quality, which serve as references for researchers.

### 2.2. Visible/Near-Infrared Spectroscopy

Visible/near-infrared spectroscopy (VIS-NIR) spectroscopy technology has extensive applications in the field of non-destructive testing for agricultural products. It irradiates the sample under test with visible light at wavelengths of 400–780 nm and near-infrared light at wavelengths of 780–2526 nm. The application of visible light spectrum technology mainly lies in its ability to capture the surface characteristics of the sample, and when the sample is exposed to near-infrared radiation, chemical bonds within functional groups such as C-H, N-H, and O-H undergo stretching and bending vibrations, leading to energy absorption. This absorption carries information about the internal quality of the sample, which is then transformed from optical signals into electrical signals via optical fibers and loaded into a spectrometer to form a spectrum. Existing experiments have demonstrated that near-infrared light within a specific range can cause almost all components to be absorbed by infrared radiation, resulting in stable spectra [20]. Non-destructive testing using spectroscopy yields a wealth of data carrying physicochemical information. However, potential defects in the spectral system may lead to baseline drift. The impact of baseline drift is manifested during the data analysis process, where abnormal changes in spectral features can introduce errors in the quantification of components in agricultural products that require precise analysis, such as moisture and sugar content. Consequently, it is crucial to eliminate noise and enhance discriminability. Mesery et al. [21] offer an exhaustive review of these methods in their article. The scientific analysis of data and the use of chemometric methods to ensure model accuracy and efficiency remain significant challenges. In the context of spectral data, partial least squares [22], support vector regression [23], and principal component regression [24] are commonly employed algorithms for dealing with quantitative regression issues. For qualitative data analysis, techniques such as principal component analysis, artificial neural networks, and Linear Discriminant Analysis [25] are frequently used for variable selection. These algorithms can be computationally efficient, especially when dealing with various types of data, as they reduce the complexity of the data without losing critical information. The synergistic application of multiple algorithms often yields improved outcomes.

### 2.3. Raman Spectroscopy

Raman spectroscopy is founded on the Raman scattering effect, which provides insights into molecular vibrations, rotations, and other low-frequency modes. When a laser irradiates the surface of a sample, the electromagnetic radiation interacts with the molecules. The majority of this light scatters elastically in a process known as Rayleigh scattering, retaining the same frequency as the incident light, which does not yield informative data. A small fraction of the light, however, undergoes a frequency shift, resulting in the Raman scattering effect. By quantifying this Raman scattering, one can glean information about the chemical bonds and functional groups at the molecular level within the sample, which is beneficial for qualitative characterization in analytical studies [26]. Raman spectroscopy is widely applied to samples across various physical states [27]. This technique provides detailed information about the chemical composition within agricultural products, making it particularly valuable for detecting internal conditions. As technology progresses, many researchers have achieved numerous successes in non-destructive testing using Raman spectroscopy. For example, in the realm of food safety, it has demonstrated effective applications in the rapid detection of pesticide residues [28], the assessment of viral infections in agricultural produce [29], and the identification of internal insect infestations in products [30]. In the context of agricultural product quality inspection, Raman spectroscopy is also extensively utilized for the detection of trace elements [31] and the authentication against adulteration [32].

### 2.4. Electronic Nose

With the ongoing advancement of sensor technology, an increasing number of studies are transferring human senses and cognitive processes to intelligent sensing systems. Electronic nose technology, an analytical technique that emulates the human olfactory system, consists of three primary functional components: sampling, gas sensors, and data analysis. These components mimic the extensive array of olfactory cells, olfactory nerves, and the brain’s neural center. By employing a multi-electrode gas sensor array to detect and identify volatile organic compounds, the sensors exhibit distinct response patterns to different gases, enabling the extraction of specific gas “fingerprint information” [33]. When the gas sample under analysis interacts with the active materials of the sensors, it triggers an instantaneous response that is converted into an electrical signal. The collective responses from multiple sensors form a response spectrum for the odor, providing a basis for effectively discriminating between different odors [34]. Based on sensor types, they can be classified into conductive sensors, sensors based on the piezoelectric effect, and other types such as optical fiber sensors and infrared gas sensors. Data analysis and processing algorithms are also central to ensuring the performance of electronic noses, primarily employing clustering algorithms, multivariate regression analysis, and neural network algorithms [35] to simulate the human brain’s recognition and analytical processes. Lu et al. [34] have provided a detailed discussion on the merits of various algorithms in their publication. The electronic nose system, known for its precise identification of characteristic gases, rapid response, and high sensitivity, is frequently utilized to assess the quality of agricultural products based on volatile odors, such as spoilage [36,37] and adulteration [38].

### 2.5. Spectral Imaging

Spectral imaging technology utilizes the sensitivity of multi-spectral channels and the varying spectral absorption rates of target objects across different bands to acquire and display images. It is a technique that combines visual imaging with spectral analysis. Visual imaging technology effectively captures the external features of samples, such as size, shape, and texture, but it cannot discern internal characteristics. Conversely, spectral technology is highly sensitive to internal quality information but does not provide information about external features. Spectral imaging technology capitalizes on the strengths of both approaches, compensating for their respective shortcomings and enhancing the throughput and reliability of detection techniques. Contrary to conventional imaging technologies that predominantly utilize visible light to acquire images, spectral imaging technology has the capability to capture images across an extensive spectrum of wavelengths, inclusive of those beyond the visible spectrum. This capability facilitates the detection of chemical and structural variations within the internal tissues of agricultural products. It has demonstrated excellent performance in detecting both internal qualities, such as sugar content, ripeness, and internal bruises [39,40], and external conditions, such as bruises and cracks [41]. Common types of spectral imaging technologies include hyperspectral imaging, multispectral imaging, and Raman spectral imaging.

### 2.6. Acoustic and Vibrating Signal Analysis

Acoustic signals are one of the more readily obtainable types of signals in nature. Agricultural products do not emit sound autonomously; hence, researchers utilize external excitation devices to induce vibrations in the samples under testing. Due to the differences in the external and internal structures of agricultural products, the response signals vary after being subjected to external excitation. By recognizing, processing to reduce noise, and analyzing these acoustic signals, a mapping relationship between acoustic characteristics and the quality of agricultural products can be established. The non-destructive testing process based on acoustic signals typically consists of three components: the excitation module, the signal acquisition module, and the signal-processing module [42]. The excitation module often employs a pendulum, which provides excitation by setting specific drop heights and angles, making it highly suitable for online detection environments. The signal acquisition module includes both contact-based equipment (such as piezoelectric sensors) and non-contact equipment (such as microphones). In terms of the signal-processing module, the Fourier transform algorithm is the most commonly used method. Detection technology based on acoustic signals has produced numerous research findings in areas such as fruit firmness [43], crispness [44], and internal mold [45], and relatively mature detection equipment has been developed in countries like The Netherlands and Japan.

## 3. Factors Affecting the Stability of Detection

**Figure 1:**
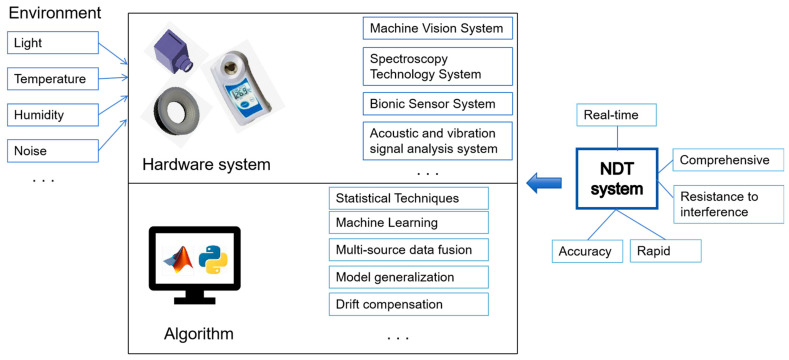
Non-destructive testing system.

The stability of non-destructive testing for agricultural product quality demands that the detection system maintains robustness and repeatability over an extended period, ensuring the efficient operation of the testing tasks. A typical non-destructive detection system can be viewed as an ensemble of detection equipment and a suite of algorithmic models for data analysis and processing, as depicted in Figure 1. During its application, the system is inevitably subjected to various sources of interference, which can lead to anomalies in detection data, a decrease in detection accuracy, or even errors, thereby impacting the overall completion of the non-destructive testing tasks. This section will enumerate and elucidate the intrinsic and extrinsic factors that affect the stability of non-destructive testing for agricultural product quality.

### 3.1. Influence of Sample Properties

The intrinsic characteristics of agricultural products are highly variable, with significant individual differences even among the same species of produce. In the actual detection process, the majority of errors often originate from the impact of the sample’s inherent properties on the detection system. Sample attributes are multifaceted and can be divided into two main categories: the physical properties and the biological properties of the samples. This section will explain and analyze the effects encountered during the detection process from these two perspectives.

#### 3.1.1. Physical Properties of the Sample

For specific agricultural products awaiting inspection, each individual sample exhibits unique physical properties, such as size, firmness, weight, skin color, and temperature. These variations can directly or indirectly impact the stability of the detection process. The uneven surface conditions, curvature, and external textural characteristics often result in non-uniform brightness distribution across the sample’s surface. In non-destructive detection models that rely heavily on machine vision technology, this can lead to image ghosting and shadowing. The presence of overlapping pigments within the sample’s skin, which affects light absorption [46], can cause differences in surface color to alter spectral absorption, thereby indirectly influencing the analysis of the sample’s internal quality. Yao et al. [47] prepared test watermelons into juice to measure the internal SSC, revealing the impact of liquid surface temperature effects on the average absorbance of near-infrared spectra. In scenarios where the determination of agricultural product quality characteristic gases is concerned, the temperature and water vapor content between samples are also significant interference factors [48]. Furthermore, for different types of agricultural products, the influence of physical properties on the model is even more pronounced. In the assessment of meat quality, the performance of prediction models for main components such as protein, moisture, and fat largely depends on the animal species. Variations in components such as moisture content, myoglobin content, fat content, and the type and structure of muscle fibers can all influence the characteristics of the meat, such as tenderness, juiciness, and flavor. Variations in components can cause changes in the predictive accuracy of models across different breeds and conditions [49]. When using near-infrared spectroscopy to assess the internal quality of fruits, the increased thickness of the fruit skin enhances the interference with light penetration, affecting light transmission characteristics and reducing the signal-to-noise ratio of spectral data. Consequently, the transmission mode of near-infrared spectroscopy must be selected differently for fruits with varying skin thicknesses. Syazwan et al. [50] also investigated the impact of the characteristics of different parts of the thick-skinned watermelon’s skin on the detection of watermelon ripeness, highlighting that measurements at different positions of the sample may yield different results. It is evident that the detection instability caused by the physical properties of the samples is primarily concentrated in the processes of data collection and algorithm processing, which increases the complexity of detection system design and poses challenges for the generalization of algorithmic models.

#### 3.1.2. Biological Properties of the Sample

Common biological characteristics of agricultural products encompass variety, season, geographical origin, and growth conditions. Due to the natural growth of these products, the biological properties of the same category can diverge into quality trait variations under different conditions such as light exposure, soil, and precipitation [51,52]. Studies focusing on the reduction in model robustness due to biological properties have predominantly centered on spectral technology. Numerous investigations into the internal quality assessment of fruits have revealed significant model deviations due to the oversight of sample biological property variations, including sample size, variety, origin, growth conditions, and fruit age [53,54,55]. From our perspective, research on the impact of biological properties on model robustness remains relatively constrained. However, the instability observed in spectral technology applications for fruits and vegetables, attributed to biological property variations, already underscores that these variations are an indispensable factor to consider when constructing models.

### 3.2. Influence of Environmental Factors

The influence of environmental factors is primarily focused in two areas. First, there is the decline in performance of the detection system’s equipment due to environmental impacts, which is predominantly reflected in the interference from external factors on the sensitivity of precision instruments. Second, there is the direct impact of environmental uncertainties on the system itself, such as the effects of ambient light and noise. This chapter will analyze the instability caused by these factors in non-destructive testing methods.

#### 3.2.1. Temperature

Whether in a laboratory setting or in the context of online detection within application scenarios, temperature is a critical external factor. Especially for equipment used in online detection environments, there can be significant variations in ambient temperatures across different settings, and the equipment must withstand prolonged operation [56]. Inevitable weather fluctuations and changes in the temperature of the samples to be inspected can adversely affect the robustness of the entire detection system. Detection models that utilize spectral technology and electronic noses are particularly vulnerable to the influence of temperature.

When employing visible/near-infrared spectroscopy for non-destructive testing, the stability of the models is predominantly influenced by the performance of two key hardware components: the light source and the optical signal detector. Greensil [56] analyzed the performance of the light source across various operating temperatures and noted that temperature significantly impacts the intensity output of the light source, introducing additional electronic noise into the system. Furthermore, an excessively high power of the light source or elevated operating temperatures can affect the temperature of the samples, potentially causing burns on the surface of the samples under test [57]. The light source detector is responsible for receiving and processing transmitted or reflected light signals; temperature fluctuations can nonlinearly affect the stability of the instrument’s wavelength, leading to changes in the detector’s output impedance [58], which in turn results in inaccuracies in the output signal. From a chemometric standpoint, temperature variations can alter the strength of hydrogen bonds in water [59], indicating that changes in temperature directly affect the measurement of water-containing substances within the samples, particularly for fruits and vegetables, which are largely composed of water. Zhang et al. [60] also investigated the impact of temperature-induced cell damage in fruits on the interaction with incident light, providing cellular-level evidence that temperature affects the stability of spectral analysis.

The influence of temperature on electronic noses is predominantly concentrated on the hardware performance of their gas sensor arrays. Electronic noses are not universally adaptable to a variety of complex detection scenarios and must function within specified temperature ranges; failure to do so can result in physical damage to the sensor arrays [61]. Temperature fluctuations can affect the reactions at the sensor surfaces, consequently impacting the detection thresholds and response times of the sensors. The existing literature has pointed out that sensors operating at different temperatures display similar performance characteristics to sensor arrays with varying doping levels [62]. These temperature differences can influence the sensors’ selectivity for gases, thereby affecting the accuracy of the detection process.

#### 3.2.2. Humidity

Humidity is generally classified into absolute humidity and relative humidity. Detection systems that utilize electronic noses and spectral technology are particularly sensitive to variations in humidity. Absolute humidity refers to the amount of water vapor present in the ambient air, whereas relative humidity is the ratio of the vapor pressure in the air to the saturation vapor pressure. This ratio indicates the proportion of the moisture content in the air to the maximum possible moisture content under the same conditions, and it is more frequently employed in the analysis conducted by electronic noses [63].

In non-destructive testing models that employ visible/near-infrared spectroscopy, changes in humidity can affect the refractive index of optical components within the instrument. This, in turn, influences the path and optical path difference that light travels through, and consequently, the absorbance of the spectrum is affected by these humidity variations [64]. When humidity levels are excessive, water vapor in the environment can absorb some of the light emitted by the light source, leading to radiation attenuation [65,66], which can cause fluctuations in the spectral output.

For electronic noses, the influence of humidity can lead to significant baseline drift in the sensors, an effect that is more pronounced than that of temperature [67]. Most electronic noses employ an array of metal oxide sensors. Due to the presence of surface reactions, when high humidity conditions prevail, water vapor enters and is ionized by sufficient potential, then adsorbed onto the surface of the metal oxide. This process reduces the number of active sites available for the target gases [68], which slows down the reaction rate of the target gases and subsequently diminishes the sensitivity of the sensors.

#### 3.2.3. Light

Non-destructive testing methods that require a light medium, such as machine vision and spectral technology, are also susceptible to the influence of light. The intensity, position, and angle of lighting can easily affect the results. Here, we specifically refer to the impact of external light sources, not the factors affecting the design of light sources within the equipment. For machine vision technology, the visual system is highly sensitive to ambient light, making it difficult to maintain consistency in the working environment, especially in outdoor inspection scenarios. Uncontrolled lighting can impose a burden on the processing process and make the results uncontrollable. Poor lighting conditions can affect the total light intensity on the surface of the object being inspected, adding background noise to the output image, which affects analysis and detection [69]. Insufficient lighting can also obscure the details of the sample being tested. In spectral technology, the selection and setup of light sources are inherently a cautious consideration, while external lighting appears in the form of interference with the spectrum. Its randomness and uncontrollability increase the difficulty of using portable spectrometers for measurements in outdoor environments [65].

#### 3.2.4. Environmental Adaptability of the Model

In the realm of non-destructive testing for agricultural products, developing a long-term, stable algorithmic model presents significant challenges. The stability of models across different agricultural products is highly sensitive to variations in growth conditions and genetic traits [55]. Furthermore, a model that is stable for a specific issue often deviates or even becomes ineffective when there are changes in measurement instruments, testing environments, or sample conditions. For example, a model developed on one spectroscopic instrument may not be applicable for estimating property values from the spectra of a different instrument; similarly, non-destructive testing that is stable in one region may fail in another. These discrepancies are usually attributed to the differences in the craftsmanship and performance parameters of the instruments, as well as the diversity of environments and samples. The samples used to build the model rarely encompass all the characteristics of the samples to be tested in the future, making it difficult to achieve a system capable of handling all tasks across every application environment. Appropriate decisions regarding the design and development of such models can only be made after specifying the requirements for a particular application. In the current pursuit of model accuracy and universality, the lack of strong environmental adaptability in models is also an unstable factor affecting the application of non-destructive testing technology, which remains a focal point of research.

### 3.3. Influence of Instruments and Equipment

A robust non-destructive testing system requires not only a stable algorithm but also instruments and equipment that are resilient to drifts induced by uncertain factors. The wear and tear of testing instruments over time, as they age, is an inevitable part of their life cycle. A comprehensive non-destructive testing setup is composed of multiple parts, and in online detection environments, it frequently incorporates various automated devices. Neglecting any aspect during the design phase can adversely affect the performance of the system. This section will analyze the factors that impact the stability of the testing from the viewpoints of both instrument performance and the soundness of the equipment’s structural design.

Non-destructive testing equipment is equipped with a variety of sensors, whose output values tend to change as the sensors age. This change is typically linear and is known as linear drift noise [70]. Sensors that have aged become less adaptable to their environment and are more vulnerable to the influence of external factors such as temperature and humidity, which can diminish the reliability of the test results. The aging of instruments is an inevitable process; at the time of manufacture, the parameters of the instruments are labeled based on the performance of the sensors, so it is imperative to use them strictly within the calibration lifespan specified by the manufacturer. Operating beyond the prescribed limits can lead to accelerated aging [56].

In non-destructive testing systems that utilize machine vision technology, the most critical component is the image acquisition device, typically employing high-definition cameras to gather visual data. The camera’s specifications, such as resolution, focus, and lens quality, are crucial factors that influence performance. The camera’s resolution and focus directly affect the clarity of the images and the ability to capture fine details; an insufficient resolution can lead to blurry images, making it challenging to detect minor defects, while an excessively high resolution might increase the data-processing load, potentially slowing down the system’s response. Incorrect focus can also result in blurred images, which can compromise the accuracy of visual inspections. The process of image acquisition and processing cannot rely solely on image-processing algorithms to meet real-time requirements [71]; the performance of the image sensor in terms of response time, dynamic range, and processing speed is also critical [72]. Commonly used light sources in machine vision systems are inevitably subject to degradation over time, such as fluorescent lights, which can experience a drop of about 15% in light energy within the first 100 h of operation, with a continuous decline in light output as usage time extends.

The performance of spectral technology is largely influenced by the performance of the instrument, particularly in two critical components: the light source and the spectrometer (detector). The light intensity from the source can fluctuate during operation due to changes in temperature, voltage, and other factors, leading to drift in the spectrometer’s baseline and the introduction of electronic noise. These variations can significantly impact the repeatability and reproducibility of the data acquired by the instrument [73]. When employing near-infrared spectroscopy, it is essential to adjust the light transmission mode according to the target being analyzed. Near-infrared spectroscopy is divided into diffuse reflection, transmission, and transflectance modes based on the mode of light transmission. The penetration capabilities differ across these modes, which is also a factor that can influence the detection outcomes.

For electronic nose systems and other gas sensors, their performance is largely determined by the sensor array and its internal configuration. The efficacy of these sensors is contingent upon materials, environmental factors such as temperature and humidity, sensor fouling, interference from other substances, and the construction of the sensors themselves [74]. In the context of non-destructive testing for agricultural products, it is essential that the collection and analysis of gases occur concurrently. A study by Scott et al. [75] elucidates how the fluid dynamics within the electronic nose’s internal chambers can affect sensor responses. Similarly, Mahdavi et al. [76] have explored how variations in gas flow rate and velocity can alter the response of gas sensors, demonstrating that the flow dynamics of the gas under test can also influence the performance of gas sensors.

In the detection and analysis of acoustic and vibration signals, the use of contact sensors in the signal reception module can alter the original vibration patterns due to direct interaction with the sample. Conversely, using non-contact collection modules, like microphones, while preventing direct contact with the sample, makes them highly susceptible to environmental noise, which in turn impacts the acquisition and analysis of the signals.

In the context of online detection, numerous unstable factors arise primarily from design shortcomings in the equipment, as it is challenging to control external conditions as effectively as in a laboratory. For example, in machine vision systems, data collection often relies on a single camera, which struggles to capture the entire surface of the sample being inspected. This difficulty in obtaining comprehensive imagery hampers the accurate calculation of defect ratios and poses challenges for grading and sorting processes [77]. The randomness of sample movement and conveyance can also result in inconsistencies in the measured area and optical path with each measurement. Additionally, the stems and leaves of fruits might obstruct the light source, and non-uniform spectral measurements can introduce instability into the results [78]. Maximizing the system’s performance requires a holistic consideration of various factors, which reflects the complexity involved in selecting appropriate instruments and designing the apparatus structure.

## 4. Methods for Improving Detection Stability

### 4.1. Optimize the Algorithm Model

#### 4.1.1. Drift Compensation Algorithm

Drift denotes the slow variation in sensor output that occurs over time. Drift compensation algorithms are utilized to counteract performance changes in instruments and equipment that arise from prolonged use due to extrinsic factors such as physical and biological alterations, environmental shifts, and the aging of devices. These algorithms are essential for preserving the stability and precision of non-destructive testing systems.

In the field of spectral technology, data preprocessing techniques are frequently utilized for drift compensation. These techniques, grounded in signal processing and statistical analysis, encompass a range of methods aimed at enhancing and correcting spectral analysis data, specifically targeting issues such as baseline drift, irrelevant factors, and noise. Commonly employed spectral data preprocessing algorithms include differentiation, continuous wavelet transform (CWT), normalization, and smoothing. Studies have shown that these preprocessing techniques can significantly improve model accuracy when dealing with the physical and biological variations present in fruits and vegetables [79]. By employing data preprocessing algorithms, the performance of models in handling samples with physical discrepancies can be enhanced. A multitude of studies on data preprocessing algorithms have shown promising results in practical applications. Zhu et al. [80] applied a Savitzky–Golay filter, which is a widely used digital filter that is particularly adept at data smoothing and reducing noise through local polynomial fitting, and standard normal variate (SNV) analysis as spectral preprocessing methods, effectively reducing noise in spectral data and mitigating spectral variations caused by surface light scattering. Roger et al. [81] introduced a preprocessing algorithm based on external parameter orthogonalization (EPO), which eliminates the most externally influenced aspects from the feature space, thereby reducing measurement accuracy biases in sugar content determination due to sample temperature variations. Gabrieli [82] employed multiplicative scatter correction (MSC) to address baseline drift in spectra resulting from physical effects, while Shyam et al. [83] used a variety of data preprocessing algorithms to minimize biological property variations in mangoes from seven different regions that could arise from geographical factors. Other researchers have targeted the different maturation seasons and growth conditions of the samples [84,85], utilizing methods such as linear regression [86] and differentiation to eliminate variations caused by external disturbances. Table 1 summarizes some research efforts where the optimization of data preprocessing algorithms has led to a reduction in spectral data variability.

In the domain of machine vision, models are required to exhibit robust adaptability to non-structured environments. This includes the capacity to withstand interference from unexpected environmental factors, random object positioning, occlusions, and lighting disturbances during the inspection of samples. Additionally, researchers must design specific correction algorithms to address variations in the physical or biological properties of the samples. Yuan et al. [87] accounted for the variability in lighting conditions and enhanced the dataset through light transformation and noise adjustment techniques. They developed a more stable cherry-tomato recognition model adaptable to various lighting conditions using an improved SSD algorithm. This strategy of compensation via data augmentation could be transferred to the application of online non-destructive testing for agricultural products. Li et al. [88] created an algorithm based on light intensity transformation for detecting surface defects on oranges, resolving issues caused by uneven light reflection due to changes in the shape of the samples being inspected. Zhu [89] introduced a 3D Shape Enhancement Transformation (SET) algorithm that compensates for the randomness in the placement and orientation of samples during detection. Ma et al. [90] have investigated the coupling effects between camera self-heating and ambient temperature on image distortionconstructed a thermically induced error compensation model that accommodates variations in both camera self-heating and ambient temperature fluctuations.Other segmentation algorithms and light intensity correction algorithms have also been developed to address the variations caused by the random shape and light intensity deviation of the samples [91,92,93]. Table 2 provides a summary of drift compensation strategies in machine vision.

For electronic noses, drift compensation is primarily aimed at mitigating the effects of environmental temperature and humidity, as well as gas flow rates, on the sensors’ detection sensitivity. Liang et al. [94] classified the causes of drift in electronic noses into three categories: operational environmental impacts, background interference, and dynamic interference. Background and dynamic interferences mainly refer to the cross-sensitivity of gas sensors in complex gas environments, which can interfere with the detection of target gases. There is extensive research on these interferences in environmental monitoring and wound detection. For non-destructive testing models of agricultural products, the focus is more on resolving interference from environmental temperature and humidity, as well as instrumental issues. Kashwan et al. [95] investigated a parameter model for sensors that calculates drift coefficients (defined as the deviation in the electronic nose response per unit change in surrounding temperature and humidity conditions) to eliminate drift in the response data captured during online testing. This approach significantly improved the clustering results for tea and spice flavors when integrated with principal component analysis (PCA) in a pattern recognition scheme. Natale et al. [96] employed Independent Component Analysis (ICA) to separate target data from environmental interference, enabling the differentiation of two types of fruits under varying temperature and humidity conditions. Liu et al. [97] developed a Multiple Overlapping Sniffing Strategy (MOSS) that continuously draws in gas, enhances dynamic airflow, and stimulates the sensor array to produce feature-rich signals. This strategy compensates for the inability of gas sensors to be exposed to airstreams for extended periods due to their non-dense and discontinuous nature.

#### 4.1.2. Model Generalization Algorithm

Building on the issue of model generalization discussed earlier, there are two prevalent approaches to enhancement: one is model transfer, which employs transfer correction algorithms such as Direct Standardization (DS) and Piecewise Direct Standardization (PDS). These methods allow for the application of well-established models in new environments, eliminating the need to train new models from scratch. The other approach is global model calibration, which involves integrating as many external variables as possible into the existing model to account for all potential factors that could affect stability, thereby ensuring the model has adequate variability. Table 3 offers a comprehensive overview of the research conducted on algorithms for model generalization.

In the field of model transfer, extensive research has been conducted by scholars over an extended period. Weng Haiyong et al. [98] employed the Direct Standardization (DS) algorithm combined with the Extreme Learning Machine (ELM) discriminant model to facilitate model transfer for the detection of citrus canker across different spectrometer models, achieving a model accuracy of 95%. Fan et al. [99] utilized the Slope and Bias (S/B) correction method in conjunction with Competitive Adaptive Reweighted Sampling (CARS) and the Successive Projection Algorithm (SPA) to determine the soluble solids content (SSC) in apples, carrying out model transfer across different years and achieving favorable evaluation metrics on the prediction set. Liu Jiao [100] employed spectral analysis to assess the water content and pH levels of pork, which are key indicators of pork quality, and explored various algorithms to accomplish model transfer among three different pork breeds. The study identified both the Piecewise Direct Standardization combined with Linear Interpolation (PDS-LI) algorithm and the Spectral and Predicted Values Synchronization (SPVS) algorithm as outstanding hyperspectral model transfer methods. Bababan et al. [101] evaluated the effectiveness of neural network algorithms and mathematical function transformations in transferring models between multiple instruments during the detection of volatile substances in milk using electronic noses, validating the feasibility of model transfer through variable correction. Sun et al. [102] applied external parameter orthogonalization (EPO) and Generalized Least Squares Weighting (GLSW) to migrate a laboratory-based spectral detection model to a portable spectrometer for field studies on the SSC of navel oranges.

Within the agricultural sector, the application of global models is prominently demonstrated in spectral technology. Fan et al. [103] constructed a global model that accounts for positional variations, thereby mitigating the biases in the soluble solids content (SSC) indices of apples attributable to location-specific factors through multi-site detection. Suo Yuting et al. [104], in their investigation of remote sensing for the quality assessment of winter jujubes in southern Xinjiang, developed inversion models for the bidirectional reflectance distribution function and moisture content of these jujubes under diverse meteorological conditions and spectral wavelengths. The objective was to eliminate the constraints of weather factors on the near-surface quality remote sensing of southern Xinjiang winter jujubes, thereby augmenting the efficiency and precision of the detection process.

When examining these two classes of model generalization algorithms, global models present a more straightforward implementation strategy and can effectively address the impacts of certain physical or biological disparities. Nonetheless, to enhance model robustness, it is imperative to introduce additional variabilities into the calibration dataset. The incorporation of exogenous data, while complicating the model, is a necessary step. Model transfer, despite its more complex algorithmic design and greater specificity compared to global models, does not require the utilization of an extensive amount of experimental data, thus diminishing the workload in data analysis and increasing the flexibility of the algorithm. The generalization of models also requires a judicious balance between universality and specificity, contingent upon the actual conditions at hand.

#### 4.1.3. Deep Learning Algorithm

Deep learning, a pivotal subset of machine learning, has experienced exponential growth in recent years, significantly advancing and disseminating artificial intelligence, thereby propelling technological evolution. Deep learning models are trained on extensive datasets and leverage substantial computational resources to automatically extract features from raw data. These models employ optimization algorithms to adjust the weights and biases within the network, thereby minimizing the loss function. This approach endows deep learning with heightened efficiency and potency in managing complex datasets. As the corpus of collected data expands and is refined, the models continue to learn and adapt to the underlying features. Concurrently, the relentless refinement of deep learning algorithms and models by researchers has led to a steady enhancement in the stability and accuracy of these algorithms, enabling them to perform an array of tasks with increasing excellence.

The advancements in deep learning have significantly bolstered the agricultural sector, with deep learning algorithm-based non-destructive detection technologies being widely implemented and leading to substantial increases in agricultural productivity. Deep learning methodologies employing visual techniques can adeptly extract image features, which are extensively utilized in the external condition assessment of agricultural produce, including the identification and detection of morphological characteristics [87,106,107]. These methodologies also possess considerable potential for the detection of external injuries and defects in agricultural produce [108,109,110]. When augmented with spectral data for training, deep learning algorithms can further be applied to assess internal quality attributes such as firmness, soluble solids content (SSC), and internal disorders [111,112,113]. By converting acoustic vibration signal analysis into sonogram images and employing deep neural networks with transfer learning, it is possible to analyze internal disease conditions in fruits [45,114]. Time-series analysis, a prevalent statistical learning approach, is employed to examine data points arranged in a temporal sequence, with the objective of forecasting future trends, seasonal patterns, cyclical variations, and other forms of long-term and short-term fluctuations based on historical data. Utilizing time-series algorithmic models allows for the estimation of postharvest shelf life and disease status in agricultural produce [115]. Through the analysis of imagery or sensor data, the grading and sorting of agricultural products can be achieved [116,117], which effectively manages the process from harvest to inventory and ultimately to the consumer, fostering sustainable development within the agricultural supply chain. Table 4 provides a comprehensive summary of various applications of deep learning technology in the domain of non-destructive detection of agricultural products in recent years.

#### 4.1.4. Data Fusion Algorithm

Multi-source data fusion algorithms are methodologies designed to synergistically integrate data from disparate sources and modalities, yielding more precise and holistic outcomes. Such algorithms are crucial for facilitating comprehensive assessment and decision-making processes, as well as for mitigating data redundancy [118]. Within the context of non-destructive detection of agricultural product quality, data acquired from a solitary detection method often falls short in terms of comprehensiveness, failing to adequately encapsulate the full spectrum of characteristics inherent to the sample under inspection. Consequently, the precision of detection results derived from such methods is frequently suboptimal. The data procured from various non-destructive detection techniques exhibit multi-source and heterogeneous characteristics. Data fusion techniques can harness the complementary nature of these features, theoretically compensating, to a certain extent, for the limitations inherent in the detection efficacy of single-type data [119]. Employing data from multiple sources facilitates a more holistic comprehension of agricultural products, offering additional insights. The integration of multiple data sources enables cross-validation of results, which in turn augments the precision, credibility, and thoroughness of the evaluation process. This approach enhances the quality of data analysis by leveraging the collective strengths of diverse data sources.

**Figure 2:**
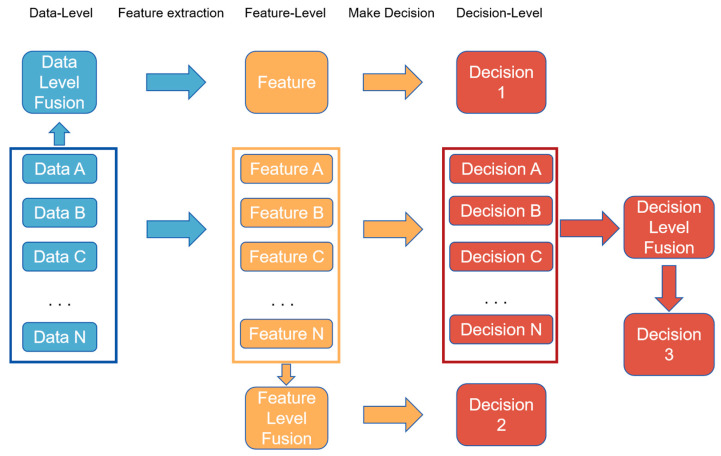
The process of the data fusion algorithm.

Data fusion, conceptually, can be stratified into three distinct levels: low-level (data-level) fusion, medium-level (feature-level) fusion, and high-level (decision-level) fusion [120]. Figure 2 delineates the methodologies of data fusion across these hierarchical strata. Each fusion strategy possesses unique strengths and weaknesses, necessitating a selection based on the specific attributes of the detection task at hand. Data-level fusion is the starting point of data fusion, involving the merging of raw data from different sensors or sources into a unified dataset. The importance of data-level fusion lies in its provision of a comprehensive data foundation for subsequent analysis, ensuring the integrity and consistency of the data. After feature extraction, feature-level fusion combines features from different data sources. It is significant that it integrates the information contained in different features, thereby offering a more comprehensive perspective to understand the data, which is crucial for improving the accuracy and depth of analysis. Decision-level fusion occurs after various independent decision-making processes; it combines these decision outcomes to produce a final decision. This step integrates judgments from different decision-making processes, reduces the bias that may come from a single decision, and enhances the accuracy and reliability of the decision. The field of multi-source data fusion algorithms is advancing in response to the escalating volume and intricacy of data. Over recent years, researchers have harnessed multi-source data fusion theories to develop a suite of non-destructive detection models for agricultural products that integrate multi-modal features, yielding superior outcomes. Hu et al. [121] utilized a visible/near-infrared and short-wave infrared hyperspectral imaging system to execute data-level fusion for the detection of prevalent pesticides on the rinds of cantaloupes, discovering that the integrated spectral data offered enhanced classification accuracy for pesticide residues compared to single-spectrum identification rates. Zou et al. [122] employed a data fusion approach combining audio analysis and near-infrared spectroscopy to assess the ripeness of watermelons, thereby enhancing the discriminative efficacy of the detection model over those relying on a single methodology. Xu et al. [123] amalgamated a visual model with near-infrared spectroscopy, employing Savitzky–Golay smoothing, genetic algorithms, convolutional neural networks, and partial least squares regression for processing and modeling. They successfully achieved favorable results in the quantification of SSC in Luogang oranges through feature fusion, markedly improving detection precision. Wang Nannan [124] constructed separate predictive models for banana chlorophyll content, firmness, and SSC predicated on spectral technology and image features. Thereafter, by integrating the predictions from these two models using a fuzzy neural network (FNN), the ultimate predictive output was ascertained; it was observed that the model fusing spectral and image data at the decision level outperformed both the standalone spectral data model and the image model in terms of accuracy. Table 5 provides a comprehensive summary of recent studies that have leveraged multi-source data fusion methodologies to augment the precision of non-destructive detection.

### 4.2. Optimize the Performance of Instrument and Equipment

The stability of the hardware performance in non-destructive testing equipment and the rationality of the equipment’s structural design are paramount for acquiring high-quality detection signals. This section will systematically review the methodologies for enhancing the hardware components of non-destructive testing systems that utilize machine vision, spectral analysis devices, and electronic noses. Additionally, it will address the refinements made to equipment deficiencies within the context of online detection environments.

#### 4.2.1. Machine Vision System

**Figure 3:**
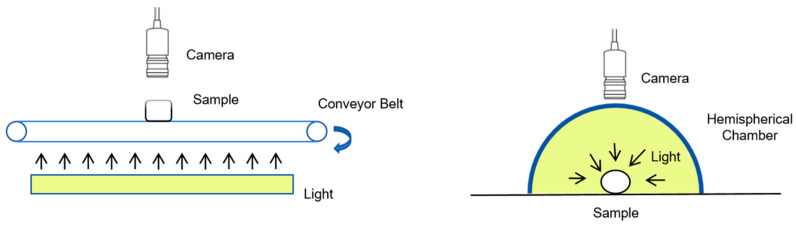
Backlight and spherical integral light source.

In the domain of machine vision technology, the image acquisition system is constituted by three essential components: light, a camera, and an object. The objective of the image acquisition system is to optimize the selection and efficient utilization of light and the camera to capture the most optimal image data of the sample under inspection. Superior lighting conditions can offer stable and uniform radiance, markedly improving the quality of captured images and alleviating the demands of subsequent image-processing tasks. Where the detection environment fails to provide sufficient illumination, artificial lighting must be strategically employed, with common configurations including front lighting, back lighting, ring lighting, strip lighting, and spherical integral lighting. As depicted in Figure 3, Blasco et al. [128] positioned fluorescent tubes beneath a transparent conveyor belt to backlight the inspected citrus segments, enhancing the contrast between the background and the sample segments, thereby facilitating image segmentation. Riquelme et al. [129] placed olives within a foam hemisphere integral sphere, which provided uniform illumination for spherical samples, circumventing issues with shadows and reflections. The requirements for lighting vary with different objects of detection and environmental conditions, mandating a case-specific selection and optimization process.

The two predominant types of image sensors currently in use are CCD (Charge-Coupled Device) and CMOS (Complementary Metal-Oxide Semiconductor). Relative to CCD, CMOS offers superior processing speed and lower power consumption, albeit with a trade-off in image quality. Advances in semiconductor technology have facilitated the integration of on-chip analog-to-digital (A/D) conversion and signal-processing capabilities, thus heralding a shift from imaging sensors to image-processing sensors [71]. Kim et al. [130] have amalgamated image–signal-processing functionalities with CMOS image sensors, enabling on-sensor operations such as low-pass filtering, velocity estimation, edge detection, and smoothing. This integration capitalizes on the high processing velocity characteristic of CMOS while concurrently diminishing the sensor’s footprint and energy consumption. Given that numerous stages of visual applications necessitate autonomous operation, intelligent cameras must possess substantial and expeditious data-processing capabilities. Embedding image-processing algorithms directly onto the image sensors enhances both the efficiency and precision of these operations.

Inspection equipment leveraging machine vision technology frequently confronts the challenge of not being able to acquire comprehensive surface information of the specimens under examination. One solution to this limitation is the employment of a multi-camera system to simultaneously capture and integrate images. Peng Yankun et al. designed a transmission mechanism utilizing a fruit cup that facilitates the collection of images from multiple perspectives. By manipulating light refraction through the transparent fruit cup, they were able to obtain full-surface imagery of the produce and introduced a method for synthesizing these images along with an algorithm for the correction of defect area measurements, effectively detecting and classifying defects on the entire surface of apples. An alternative approach involves the autonomous rotation of the specimen. Zhang et al. [131] set up an inspection line with a conveyor belt equipped with a rotating drum, where apples to be inspected are positioned between two sets of drums. As the drums revolve around their axis, cameras can secure images from various angles during the image acquisition process, thereby acquiring full-surface information for defect detection. Baneh et al. [132], in their comprehensive review, elaborated on the design philosophies of mechatronic components integrated into machine vision models for the grading and sorting of apples, offering a blueprint for the design of other machine vision-assisted systems for the categorization of agricultural produce.

#### 4.2.2. Spectral Detection System

Within the domain of enhancing spectroscopic device performance, researchers have concentrated on refining the light source. The stability of the light source is a critical factor influencing both the stability of the instrument and the precision of analytical results. Utilizing a “temperature regulation plus constant current” approach can effectively manage the stability of the light source; this method involves powering the light source with a precise constant current source to stabilize the output optical power. However, it necessitates the use of a specialized cooling system for temperature control, which can be impractical. Zhou Xiaoli et al. [133] introduced the concept of an optical power negative-feedback control system and designed a corresponding circuit. The core idea was to counteract any instability in optical power caused by various factors by adjusting the terminal voltage of the light source to maintain a stable optical power output, with the system reaching a stable state within approximately 15 min after startup. Xu Huirong et al. [134] proposed a device that integrates an automatic reference with a shutter mechanism, controlled by a servo motor that manipulates a barrier. This innovation allows for the collection of dark environment data without the need to extinguish the light source, thus preventing physical damage to the optical fibers that could result from direct exposure to the light source in the absence of samples within the detection environment. In the application of spectral technology (as well as machine vision), it is advisable to employ a standardized set of lighting parameters and conditions to ensure the consistency and repeatability of data acquisition. Nevertheless, the determination of lighting parameters, including light intensity, the quantity of light sources, and their spatial arrangement, necessitates rigorous testing and a process of optimization to select the most suitable configurations.

Systems using spectroscopic technology for online detection similarly confront the impact of sampling variations due to the position of the sample under inspection and the conditions of sample loading. Wang et al. [135] utilized a posture adjustment device to correct the orientation of apples during the loading process, thereby avoiding discrepancies in the optical path that could affect the outcomes of spectral analysis. Jie et al. [136] developed a prototype system for online spectral detection, tested and refined the conveyance speed, and optimized the accuracy of determining the soluble solids content (SSC) in watermelons, leading to more precise sorting. The device proposed by Xu Huirong et al. can, in the context of online detection, not only acquire spectral information about the internal quality of fruits but also automate the collection of reference spectra through the automatic placement of references, thereby enhancing the level of automation and the precision of detection.

#### 4.2.3. E-Nose System

Within the context of agricultural product inspection, augmenting the quantity of gas sensors can incrementally improve gas selectivity. However, an indiscriminate increase in sensor numbers can escalate the complexity of system design and, potentially, exacerbate drift phenomena under external interference. Zhang et al. [137] conducted an optimization of the electronic nose array. They utilized support vector machines (SVM) and loading analysis to ascertain the most effective configuration. Furthermore, employing random forests, they ranked the significance of superior feature subsets, thereby deriving the optimal feature set. This array optimization approach enhances the reliability of the electronic nose system in detection tasks.

In the preceding discourse, we noted that electronic noses exhibit extreme sensitivity to environmental temperature and humidity, with their performance potentially declining sharply under the perturbation of extrinsic environmental factors. To curb environmental interference and ensure performance stability, researchers have investigated a range of hardware compensation strategies.

1. Surface Engineering: The capacity of gas sensor arrays to withstand temperature and humidity fluctuations is contingent upon the material properties of the sensors. Surface engineering techniques, which encompass doping and material modification, are employed to diminish the sensors’ sensitivity to these environmental parameters [138,139].
2. Physical Isolation: This approach entails the use of materials that do not adversely affect sensor performance to create a barrier around the sensor array. Examples include polylactic acid (PLA) films [140] and aluminum oxide (Al_2_O_3_) nanofilms [141], which obstruct water molecules while permitting the passage of gas molecules [142].
3. Sampling Treatment: The regulation of the sampled gas’s humidity through the application of filters or desiccants, or the incorporation of pre-concentrators during the sampling phase [143], and the implementation of treatments to control the temperature and humidity of the samples to suitable conditions prior to electronic nose analysis.

It is important to note that the hardware optimizations currently undertaken for electronic noses are predominantly employed in settings with complex gas samples, such as environmental gas detection, where the compensation strategies are specifically tailored to the target gases and lack generalizability. Nonetheless, these research endeavors and compensation strategies offer valuable insights for hardware compensation in the field of agricultural product detection.

## 5. Conclusions and Prospects

### 5.1. Conclusions

In recent years, with the progressive maturation of non-destructive testing technologies, researchers have shifted their focus from merely executing non-destructive testing tasks for agricultural products to investigating the stability of these testing systems, endeavoring to identify factors influencing system stability and to develop methods for enhancing it. This comprehensive review outlines potential instability factors inherent in commonly employed non-destructive testing methods for agricultural products and, drawing on current research, encapsulates strategies to augment the stability of such testing systems. It is evident that technologies such as machine vision, spectroscopy, electronic noses, and acoustic vibration signal analysis are extensively employed in the quality assessment of agricultural products. Detection systems leveraging these technologies are subject to influences from the physical and biological attributes of the samples, susceptible to environmental interferences, and may also exhibit instability due to the performance of the instruments and the design of the equipment, resulting in decreased detection efficiency and biased outcomes. To mitigate these interfering factors and to ensure that non-destructive testing systems maintain their robustness and repeatability over an extended period, researchers have proposed targeted compensation strategies based on performance metrics of the instruments. At the algorithmic model level, they have employed more directed feature engineering for data and further-refined processing procedures to compensate for drift. Additionally, they have suggested model generalization through model transfer and the construction of global models to improve the models’ adaptability to various environments and their universal applicability. With the emergence and advancement of disciplines such as artificial intelligence and data science, non-destructive testing models for agricultural products have been reinforced through extensive technological integration, introducing novel perspectives for enhancing the stability of non-destructive testing. Non-destructive testing model architectures predicated on deep learning technology are innovative, with algorithms exhibiting superior performance; techniques such as data augmentation and transfer learning have bolstered model performance and robustness, enabling them to address complex tasks with greater precision and velocity; multi-source data fusion has ameliorated the limitations of singular detection methods, and the processing of multi-modal data has elevated the models’ decision-making capabilities. Deep learning and multi-source data fusion algorithms demand high-quality data and entail a profound comprehension and strategic selection of features pertinent to the quality assessment of agricultural products. Moreover, these algorithms are computationally intensive, posing significant challenges that are currently being addressed through continuous research and development. This ongoing effort is expected to progressively enhance the stability and robustness of non-destructive testing algorithms, thereby improving their performance. The inception and evolution of non-destructive testing models have introduced innovative approaches to the postharvest quality inspection and grading of agricultural products, safeguarding the stability of the agricultural product supply chain. The incremental enhancement of detection stability has fortified the role of non-destructive testing technology in agricultural applications, establishing it as a significant branch within the evolution of digital agriculture.

### 5.2. Challenges and Prospects

To satisfy consumer demand for high-quality agricultural produce and to augment the profitability of agriculturalists and vendors, the advancement of non-destructive testing technologies for assessing agricultural product quality is set to persist. Beyond the current challenges, potential issues and prospective research directions may be delineated as follows:

1. Currently, we have established methods for assessing the internal quality of agricultural products. However, a comprehensive understanding of the intrinsic correlations and mutual influences among various physicochemical properties has not yet been achieved. Similarly, the etiological factors behind a significant portion of the diseases affecting agricultural product quality remain elusive. This gap underscores the need for researchers to delve into the mechanisms underlying the physicochemical properties of agricultural products. Such analyses are instrumental in enhancing our comprehension of the patterns and trends of quality fluctuations in agricultural products. They also facilitate the development of temporal models for postharvest changes in internal quality, the assessment of disease incidence, and the determination of shelf life. Rational control over the processes of harvesting, storage, and transportation is crucial for augmenting the quality of agricultural products.
2. Non-destructive testing technology, by its nature, emulates the human inspection process to evaluate sample properties. However, there can be a divergence between the evaluations derived from algorithmic systems and those of human sensory perception, particularly in environments where large-scale testing is conducted, potentially leading to model performance degradation. The integration of multi-source data fusion technology, which considers multimodal sample indicators, raises the question of whether human qualitative judgments can be quantified and integrated into model analyses. This approach would synthesize the outcomes of various instrumental measurements while also incorporating human discernment, presenting a direction ripe for extensive research.
3. The transition of non-destructive testing technology from the laboratory to practical production settings is not without challenges. Equipment parameters and structures that perform optimally under controlled laboratory conditions often encounter issues when adapted to online detection environments. Moreover, many non-destructive testing technologies, constrained by their operational principles and instrumental limitations, are ill-suited for online detection scenarios. This necessitates continuous refinement of non-destructive testing instruments and equipment structures. When selecting instruments and designing equipment, it is imperative to consider the object of detection holistically and to adjust factors such as sample conveyance speed, sample placement, and environmental indicators to achieve optimal signal acquisition and detection outcomes. This remains a complex issue that requires attention.
4. With the global agricultural development progressing towards intelligence, there is an ongoing optimization of the mechanisms for the transportation of agricultural products from the field to the consumer. Within this broader context, there is an acute necessity for a comprehensive and integrated intelligent system that encompasses the entire spectrum of activities, from the harvesting of agricultural produce to quality inspection and ultimately to sorting and packaging. This system is designed not only to economize on labor but, more significantly, to augment operational efficiency. The realization of such a system demands interdisciplinary research endeavors that capitalize on the technological strengths of various fields to address a wider array of challenges within the smart agriculture landscape.

In the prospective domain of non-destructive testing for agricultural product quality, the emphasis for researchers will increasingly shift towards achieving more robust hardware performance, enhancing algorithmic sophistication, and developing more sophisticated automated detection apparatuses. In the realm of hardware, the ongoing optimization of sensor capabilities is imperative to mitigate the impact of inevitable external interferences. Regarding data analysis and processing algorithms, the evolution of computer science suggests that deep learning, in conjunction with multimodal analysis, and the strategic acquisition and utilization of multi-source data, will emerge as focal points of research. The trajectory towards automation in the design of testing equipment is unmistakable, and the tailoring of equipment configurations to the specificities of the testing subjects and environments remains a critical area requiring resolution. The research on non-destructive testing of agricultural products is also extending its reach into the realms of postharvest quality control and processing systems, thereby fueling advancements in the study of autonomous agricultural systems.

## Figures and Tables

**Table 1:** Summary of drift compensation correction in spectral techniques.

Factors	Spectral Techniques	Applications	Target of Detection	Algorithm	Result	Reference
Shape of the sample	HSI	Measuring the distribution of hardness	Peach	SNV, Savitzky–Golay Filter	${AB\_RMSE}_{PLSR}$ = 0.711 (16%) ^1^ ${AB\_RMSE}_{LS\_SVM}$ = 2.025 (38%)	[80]
Temperature of the sample	VIS-NIR	Measuring the sugar content (SC)	Apple	EPO-PLS	Bias < 0.3° Brix	[81]
Temperature of the sample	NIR	Measuring the crude protein content	Corn	Linear regression	R = 0.910 ^2^	[86]
Origin of the sample	NIR	Measuring the PH and total soluble solid (TSS)	Mango	Multivariate scattering correction	$R_{TSS}$ = 0.762 $R_{PH}$ = 0.703	[83]
Variety and ripening season of the sample	NIR	Measuring the Soluble Solids Content (SSC)	Pepper	Second derivative, Savitzky–Golay Filter	$R_{SSC}$ = 0.87 ${SECV}_{SSC}$ = 0.59 ^3^ ${RPD}_{SSC}$ = 2.08 ^4^	[84]
Variety and origin of the sample	NIR	Measuring the Soluble Solids Content (SSC)	Cucumis melo	Second derivative	/	[54]
Variety and ripening stage of the sample	NIR	Measuring the sugar content and organic acids	Apricot	SNV	$R_{sugar}$ ϵ (0.81, 0.86)$r_{TA}$ = 0.88	[85]

^1^ AB_RMSE: the absolute difference between the RMSEC and RMSECV. ^2^ R: Multiple Correlation Coefficient. ^3^ SECV: Standard Error of Cross Validation. ^4^ RPD: Relative Percent Difference.

**Table 2:** Summary of drift compensation correction in machine vision technology.

Factors	Applications	Target of Detection	Algorithm	Result	Reference
Light, occlusion from other objects	Object detection	Cherry tomato	Improved SSD	Average Precision = 98.85%	[87]
Shape of the sample	Surface defect detection	Orange	Light intensity transformation algorithm	Detection Rate = 98.9%	[88]
Shape of the sample	Surface defect segmentation	Navel orange	Mask and edge gray-value compensation algorithm	Segment Rate ϵ (79.5%, 100%)	[91]
Shape of the sample	Defect detection	Apple	Light intensity transformation algorithm, image segmentation	/	[93]
Randomness of the placement state	Identification of stem and calyx of fruit	Apple	Three-dimensional shape enhancement	Accuracy=93.97%	[89]
Light, differences in the surface	Surface defect segmentation	Apple	Pixel sorting and threshold segmentation	/	[92]

**Table 3:** Summary of model generalization algorithm.

Methods	Differences	Applications	Target of Detection	Algorithm	Reference
Model transfer	Different spectrometers	Detecting the Citrus canker disease	Citrus	DS +ELM ^1^	[98]
Model transfer	Different years	Measuring the Soluble Solids Content (SSC)	Apple	S/B + CARS ^2^+ SPA ^3^	[99]
Model transfer	Different varieties	Measuring the water content and PH	Pork	PDS-LI ^4^ + SPVS ^5^	[100]
Model transfer	Different E-noses	Detection of volatile substances	Milk	Mathematical function transformation neural network	[101]
Model transfer	Different spectrometers, Different environments	Measuring the Soluble Solids Content (SSC)	Navel orange	EPO + GLSW ^6^	[102]
Global model	Different measuring positions	Measuring the Soluble Solids Content (SSC)	Apple	/	[103]
Global model	Different weathers, Different light intensity	Measuring the water content	Winter jujube	/	[104]
Global model	Different varieties, Different seasons	Measuring the Soluble Solids Content (SSC)	Japanese Plums	/	[105]

^1^ DS: Direct Standardization, ELM: Extreme Learning Machine. ^2^ S/B: Slope and Bias correction, CARS: Competitive Adaptive Reweighted Sampling. ^3^ SPA: Successive Projection Algorithm. ^4^ PDS-LIL: Piecewise Direct Standardization combined with Linear Interpolation. ^5^ SPVS: Spectral and Predicted Values Synchronization. ^6^ EPO: External Parameter Orthogonalization, GLSM: Generalized Least Squares Weighting.

**Table 4:** Summary of deep learning algorithms.

Methods	Applications	Target of Detection	Algorithm	Result	Reference
Machine vision	Appearance detection	Cherry tomato	Improved SSD	/	[87]
Machine vision	Appearance detection	Green peach	Attention + CNN	Precision = 97.3%, Recall = 99.7%, f1 = 98.5%,	[107]
Machine vision	Defect detection	Potato	SSD, R-CNN, RFCN	${ACC}_{SSD}$ = 92.5%, ^1^ ${ACC}_{RFCN}$ = 95.6%, ${ACC}_{RCNN}$ = 98.7%	[109]
Machine vision	Defect detection	Green plums	VGGNet	Precision = 93.8%, f1 = 0.97	[110]
Machine vision	Internal disorder detection	Apple	3D-CNN	ACC = 96% ± 1%, Precision = 98% ± 4%	[112]
HSI	Measuring the Soluble Solids Content (SSC) and firmness	Tomato	ResNet	R^2^ = 0.901, MSE = 0.018 ^2^	[111]
Raman spectroscopy	Identification of pesticide residues	Tea	CNN	Precision = 100%, ACC = 100%	[113]
Acoustic and Vibrating Signal	Internal mildew detection	Apple	CNN + SVM	ACC = 99.63%	[45]
Acoustic and Vibrating Signal	Granulation disease detection	Jelly orange	ResnetTransformer	Precision > 99% f1 > 99%Recall > 99%	[114]

^1^ ACC: Accuracy. ^2^ MSE: Mean Squared Error.

**Table 5:** Summary of data fusion algorithms.

NDT Methods	Target of Detection	Fusion Level	Algorithm	Reference
Spectral technology	Different pesticide residues on Hami melon surface	Data Level	CNN	[121]
Spectral technology, Acoustic signal	Watermelon maturity	Data Level	KNN, LDA, BP-ANN	[122]
Spectral technology, Machine vision	TSSC and water content Detection in Luogang Orange	Feature Level	SG + GA + CNN+ PLSR	[123]
Electronic nose, Electronic tongue	Discrimination of bulbus of Fritillaria	Feature Level	PCA + DFA	[125]
Electronic nose, Machine vision	Tomato maturity detection	Feature Level	LS-SVM	[126]
Spectral technology, Machine vision	Classification of banana quality	Decision Level	FNN	[124]
Spectral technology, Electronic nose	Identification for guava mechanical damage	Decision Level	LDA, FCM	[127]

## Data Availability

No new data were created or analyzed in this study. Data sharing is not applicable to this article.
